# Supplementary material for: Transcriptome Analysis Reveals Vimentin-Induced Disruption of Cell–Cell Associations Augments Breast Cancer Cell Migration
Source: Cells. 2022 Dec 13;11(24):4035. doi: 10.3390/cells11244035 (PMC9776984; doi:10.3390/cells11244035)
Supplement: Supplementary file 1 [file cells-11-04035-s001.zip › DEGs and enrichment analysis.zip!/DEGs and enrichment analysis/WT_VIMvsVector KEGG/GSEA/WT_VIM/2_OXOCARBOXYLIC_ACID_METABOLISM/2_OXOCARBOXYLIC_ACID_METABOLISM(HSA01210).html]

Details for gene set 2\_OXOCARBOXYLIC\_ACID\_METABOLISM(HSA01210)[GSEA]

|  || Dataset | fpkm.sample |
| Phenotype | sample.cls |
| Upregulated in class | WT\_VIM |
| GeneSet | 2\_OXOCARBOXYLIC\_ACID\_METABOLISM(HSA01210) |
| Enrichment Score (ES) | 0.6950812 |
| Normalized Enrichment Score (NES) | 1.1596816 |
| Nominal p-value | 0.0 |
| FDR q-value | 0.30374962 |
| FWER p-Value | 0.498 |
Table: GSEA Results Summary

  

Fig 1: Enrichment plot: 2\_OXOCARBOXYLIC\_ACID\_METABOLISM(HSA01210)      
 Profile of the Running ES Score & Positions of GeneSet Members on the Rank Ordered List

  

| PROBE | DESCRIPTION (from dataset) | GENE SYMBOL | GENE\_TITLE | RANK IN GENE LIST | RANK METRIC SCORE | RUNNING ES | CORE ENRICHMENT || 1 | ENSG00000233719 | GOT2P3 |  |  | 176 | Infinity | -0.0027 | Yes |
| 2 | ENSG00000235371 | AL122018.1 |  |  | 443 | Infinity | -0.0068 | Yes |
| 3 | ENSG00000273654 | AC020904.2 |  |  | 4160 | 2.890 | 0.0240 | Yes |
| 4 | ENSG00000161653 | NAGS |  |  | 5021 | 2.392 | 0.0869 | Yes |
| 5 | ENSG00000182054 | IDH2 |  |  | 5915 | 2.058 | 0.1385 | Yes |
| 6 | ENSG00000105552 | BCAT2 |  |  | 5931 | 2.052 | 0.2048 | Yes |
| 7 | ENSG00000235485 | AL021877.1 |  |  | 6168 | 1.992 | 0.2653 | Yes |
| 8 | ENSG00000067829 | IDH3G |  |  | 6496 | 1.924 | 0.3222 | Yes |
| 9 | ENSG00000101365 | IDH3B |  |  | 6527 | 1.918 | 0.3838 | Yes |
| 10 | ENSG00000120053 | GOT1 |  |  | 8029 | 1.619 | 0.4109 | Yes |
| 11 | ENSG00000100412 | ACO2 |  |  | 8076 | 1.613 | 0.4623 | Yes |
| 12 | ENSG00000062485 | CS |  |  | 8325 | 1.574 | 0.5092 | Yes |
| 13 | ENSG00000109576 | AADAT |  |  | 10517 | 1.316 | 0.5147 | Yes |
| 14 | ENSG00000125166 | GOT2 |  |  | 10717 | 1.292 | 0.5533 | Yes |
| 15 | ENSG00000255182 | AC084125.2 |  |  | 11073 | 1.258 | 0.5880 | Yes |
| 16 | ENSG00000167701 | GPT |  |  | 11128 | 1.254 | 0.6277 | Yes |
| 17 | ENSG00000243989 | ACY1 |  |  | 11504 | 1.227 | 0.6611 | Yes |
| 18 | ENSG00000166123 | GPT2 |  |  | 11800 | 1.201 | 0.6951 | Yes |
| 19 | ENSG00000122729 | ACO1 |  |  | 14880 | 0.977 | 0.6746 | No |
| 20 | ENSG00000234115 | AL138878.2 |  |  | 16413 | 0.893 | 0.6777 | No |
| 21 | ENSG00000060982 | BCAT1 |  |  | 17135 | 0.849 | 0.6930 | No |
| 22 | ENSG00000138413 | IDH1 |  |  | 24628 | 0.388 | 0.5788 | No |
| 23 | ENSG00000231900 | GOT2P1 |  |  | 33173 | NaN | 0.4345 | No |
| 24 | ENSG00000233634 | GOT2P5 |  |  | 33771 | NaN | 0.4247 | No |
| 25 | ENSG00000225017 | AP004289.1 |  |  | 39741 | NaN | 0.3241 | No |
| 26 | ENSG00000114786 | ABHD14A-ACY1 |  |  | 40751 | NaN | 0.3073 | No |
| 27 | ENSG00000214745 | AL391419.1 |  |  | 41116 | NaN | 0.3015 | No |
| 28 | ENSG00000228872 | AC096664.1 |  |  | 42602 | NaN | 0.2767 | No |
| 29 | ENSG00000264236 | AC096708.1 |  |  | 42636 | NaN | 0.2764 | No |
| 30 | ENSG00000225477 | AC099730.1 |  |  | 43607 | NaN | 0.2604 | No |
| 31 | ENSG00000232392 | AC002366.1 |  |  | 48848 | NaN | 0.1720 | No |
| 32 | ENSG00000216352 | AL590824.1 |  |  | 52187 | NaN | 0.1159 | No |
| 33 | ENSG00000241218 | AC063944.2 |  |  | 54966 | NaN | 0.0692 | No |
| 34 | ENSG00000230849 | GOT2P2 |  |  | 55251 | NaN | 0.0647 | No |
| 35 | ENSG00000228367 | AC108120.1 |  |  | 56934 | NaN | 0.0366 | No |
| 36 | ENSG00000272917 | AC010186.4 |  |  | 57190 | NaN | 0.0326 | No |
| 37 | ENSG00000256450 | AC007552.1 |  |  | 58514 | NaN | 0.0105 | No |
Table: GSEA details [plain text format]

  

Fig 2: 2\_OXOCARBOXYLIC\_ACID\_METABOLISM(HSA01210)      
 Blue-Pink O' Gram in the Space of the Analyzed GeneSet

  

Fig 3: 2\_OXOCARBOXYLIC\_ACID\_METABOLISM(HSA01210): Random ES distribution      
 Gene set null distribution of ES for **2\_OXOCARBOXYLIC\_ACID\_METABOLISM(HSA01210)**
